# Methanol Poisoning Without Typical Diagnostic Clues or Standard Antidotes: A Case Report on Clinical Decision-Making

**DOI:** 10.7759/cureus.90690

**Published:** 2025-08-21

**Authors:** Yusuke Miyazaki, Hiroki Takeda, Tetsuya Takahashi

**Affiliations:** 1 Emergency and Critical Care Medicine, Konan Medical Center, Kobe, JPN; 2 Emergency Medicine, Konan Medical Center, Kobe, JPN

**Keywords:** absence of visual symptoms, clinical decision-making, empirical treatment, ethanol therapy, limited health resources, methanol poisoning, normal anion gap, renal replacement therapy, toxic alcohol ingestion, urine analysis

## Abstract

Methanol poisoning is a potentially life-threatening condition characterized by severe metabolic acidosis and visual disturbances. Early diagnosis may be challenging due to nonspecific symptoms and limited access to diagnostic tools or antidotes in some settings. We report the case of a 43-year-old woman who ingested a large volume of methanol along with flunitrazepam. On presentation, she showed no signs of metabolic acidosis or visual impairment, and her anion gap remained within the normal range. Laboratory confirmation of methanol exposure was unavailable at the time. Despite the absence of typical diagnostic markers, empirical treatment with continuous renal replacement therapy and enteral ethanol infusion was initiated based on clinical suspicion. A retrospective analysis of a urine sample later revealed a markedly elevated methanol concentration. This case underscores the importance of early clinical decision-making and demonstrates that successful intervention is possible even in resource-limited environments without access to standard diagnostic or therapeutic modalities.

## Introduction

Methanol poisoning is a life-threatening condition resulting from the ingestion of methanol-containing substances, typically leading to severe metabolic acidosis, visual disturbances, and neurological damage [1]. Early diagnosis and prompt intervention are critical to prevent irreversible complications or death. Conventionally, diagnostic approaches involve detecting elevated anion gap (AG) metabolic acidosis, measuring serum osmolal gap (OG), and confirming methanol blood levels. Formic acid, the toxic metabolite of methanol, is responsible for most of the end-organ damage [2].

However, real-world clinical settings, particularly outside tertiary care centers, often encounter significant diagnostic and therapeutic challenges. Many institutions, especially smaller or regional hospitals, may lack the capacity to promptly measure serum osmolality or methanol levels, and fomepizole, an effective but expensive antidote, may not be readily available [3]. Previous case series and reviews have emphasized the importance of early intervention but largely assume access to comprehensive diagnostic tools and antidotal therapy [4,5]. Reports on methanol poisoning cases diagnosed and managed without these resources remain scarce [6].

Here, we present a unique case of methanol poisoning that was successfully managed under substantial resource limitations. Despite the absence of diagnostic markers such as AG widening, OG calculation, or methanol blood level measurement, and without access to fomepizole, treatment was initiated based solely on clinical suspicion derived from the patient's history. Remarkably, the patient survived without significant neurological sequelae. This case highlights the crucial role of early clinical decision-making and suggests that life-saving interventions are possible even in resource-constrained environments.

## Case presentation

A 43-year-old woman with a history of bipolar disorder and adjustment disorder was found staggering in a public location and brought to the emergency department (ED) by emergency medical services. On arrival, she was drowsy but arousable (Glasgow Coma Scale (GCS) E4V5M6), with stable vital signs including blood pressure of 123/89 mmHg, heart rate 66 bpm, respiratory rate 17/min, temperature 35.9°C, and SpO_2_ 97% on room air. Pupils were 5 mm bilaterally with brisk light reflexes.

Upon further questioning, the patient admitted to intentionally ingesting 200-250 mL of a solution containing 95% methanol and 5% ethanol, purchased online and mixed with juice. She also reported taking approximately 80 tablets of flunitrazepam (2 mg per tablet; total about 160 mg), which represents a massive amount with a potential risk of respiratory depression. The ingestion occurred approximately 1.5 hours before ED arrival.

Initial blood tests and venous blood gas analysis, performed shortly after arrival, revealed a pH 7.296, base excess (BE) +3.1 mmol/L, and an AG of 8.3 mmol/L (Table 1).

**Table 1:** Initial laboratory findings on admission * Measured retrospectively from a urine sample obtained on admission. JCS: Japan Coma Scale; PvCO_2_: partial pressure of carbon dioxide in venous blood; HCO_3_⁻: bicarbonate

Parameter	Value	Reference Range	Unit
Glasgow Coma Scale	E4V5M6	E4V5M6 = Normal	-
Level of consciousness (JCS)	I-1	I-1 = Alert	-
Blood pressure	123/89	90-140/60-90	mmHg
Pulse rate	66	60-100	beats/min
Respiratory rate	17	12-20	breaths/min
Oxygen saturation (SpO_2_)	97	≥94	%
Body temperature	35.9	36.1-37.2	°C
Pupils	5/5 mm, brisk light reflex	2-5 mm, brisk light reflex	-
Venous blood gas (VBG)			
pH	7.296	7.31-7.41 (venous)	-
PvCO_2_	58.0	40-52 (venous)	mmHg
HCO_3_⁻	27.4	22-26	mmol/L
Base excess	3.1	-2 to +2	mmol/L
Serum electrolytes			
Sodium (Na^+^)	139	135-145	mmol/L
Chloride (Cl^-^)	103	98-107	mmol/L
Potassium (K^+^)	3.9	3.5-5.0	mmol/L
Anion gap	8.6	8-16	mmol/L
Urinary methanol concentration*	1370	-	mg/dL

Serum osmolarity and serum methanol levels could not be measured at the facility. A urine sample was submitted for methanol analysis, but the result was not immediately available. Based solely on the ingestion history and clinical suspicion of methanol poisoning, treatment was initiated.

Two hours after ED arrival, a nasogastric tube was placed, and gastric contents were aspirated, revealing blue-colored, granular material. The patient was admitted to the intensive care unit (ICU) shortly thereafter. On ICU admission, arterial blood gas analysis revealed severe respiratory acidosis (pH 7.187, pCO_2_ 71.1 mmHg, HCO_3_⁻ 25.9 mEq/L) with preserved oxygenation (pO_2_ 265 mmHg without supplemental oxygen), suggesting hypoventilation due to central respiratory depression. Continuous renal replacement therapy (CRRT) was started approximately 3.5 hours after ED arrival and continued for 27 hours. Ethanol infusion therapy was initiated about 5.5 hours after arrival via the nasogastric route. A solution was prepared by diluting 115 mL of anhydrous ethanol with 150 mL of water (total volume 265 mL, 43%). The patient received a 50 mL loading dose followed by a maintenance infusion at 10 mL/h. Oral folinic acid (5 mg, four tablets per dose) was administered twice daily as an adjunct therapy.

**Figure 1:**
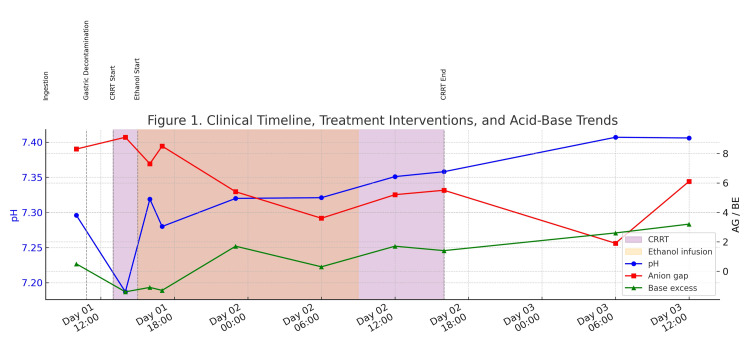
Clinical timeline, treatment interventions, and acid-base trends This figure illustrates the serial changes in arterial pH (blue), anion gap (AG) (red), and base excess (BE) (green) in a patient with methanol poisoning. The shaded orange area represents the period of ethanol infusion therapy (from day 1, 15:00 to day 2, 09:00), and the shaded purple area denotes the duration of continuous renal replacement therapy (CRRT) (from day 1, 13:00 to day 2, 16:00). Vertical dashed lines indicate key clinical events: - Ingestion (day 1, 07:30) - Gastric decontamination via NG tube (day 1, 10:50) - CRRT initiation (day 1, 13:00) - Ethanol infusion start (day 1, 15:00) - CRRT termination (day 2, 16:00)

Throughout her ICU stay, the patient remained hemodynamically stable with no neurological deterioration or visual symptoms. Serial blood gas measurements showed no progression of acidemia, with gradual normalization of pH, BE, and AG, as presented in Figure 1.

Ethanol infusion was discontinued on hospital day 2, 24 hours after initiation, due to the absence of metabolic acidosis or visual complaints. CRRT was terminated later the same day.

On hospital day 3, the patient was transferred to a psychiatric hospital for further care. She was discharged without any neurological or visual sequelae. Retrospective analysis of the urine sample collected on admission revealed a methanol concentration of 1370 mg/dL, confirming the diagnosis.

## Discussion

Methanol poisoning is a life-threatening condition characterized by the accumulation of formic acid, which leads to severe metabolic acidosis, visual disturbances, and potentially fatal outcomes [1]. However, early diagnosis is often challenging due to non-specific initial symptoms and limited access to confirmatory testing. In many clinical settings, serum methanol levels and OG or AG measurements are not readily available or may remain within normal ranges in the early phase, delaying appropriate treatment [2].

A summary of previously reported cases is provided in Table 2.

**Table 2:** Comparison of reported methanol poisoning cases AG: anion gap; OG: osmolal gap; HD: hemodialysis; CRRT: continuous renal replacement therapy; EtOH: ethanol; MeOH: methanol; N/A: not available

No.	Authors (Year)	Country	Age/Sex	Exposure Route	Serum MeOH Level	Urine MeOH Level	AG, OG	pH	Treatment	Visual Outcome	Final Outcome
1	Hovda et al., 2007 [4]	Norway	63/M	Ingestion	750 mg/L	612 mg/L	AG: 32, OG: N/A-	7.13	HD + fomepizole	Visual loss	Died
2	Epker et al., 2010 [5]	Netherlands	26/M	Ingestion	4400 mg/L	Not measured	AG: 39, OG: 73	6.69	CRRT + EtOH	Not assessable (coma)	Brain death
3	Nurieva and Kotikova, 2015 [7]	Czech Republic	33/M	Ingestion	806 mg/L	Not measured	AG: 23, OG: N/A	7.28	HD + EtOH	None	Survived
4	Nazir et al., 2016 [8]	USA	37/F	Ingestion	2370 mg/L	Not measured	AG: 29, OG: 115	6.72	HD + fomepizole	Visual loss	Survived
5	Ono et al., 2017 [3]	Japan	21/M	Ingestion	Not measured	Not measured	AG: 10.3, OG: 120	7.4	Fomepizole only	None	Survived
6	Yoshida et al., 2019 [6]	Japan	50/M	Ingestion	916 mg/L	Not measured	AG: 27.8, OG: N/A	6.83	HD + fomepizole	None	Survived
7	Balodis et al., 2024 [9]	Latvia	44/F	Ingestion	Not measured	Not measured	AG: N/A, OG: N/A	<7.0	Supportive care	Visual loss	Died
8	Current case (2025)	Japan	43/F	Ingestion	Not measured	1370 mg/L	AG: 8.3, OG: N/A	7.296	CRRT + EtOH	None	Survived

Most patients presented with markedly elevated serum methanol levels, widened AG or OG, and varying degrees of visual or neurologic impairment [3-9]. Notably, urine methanol levels were rarely reported. In contrast, our case lacked typical diagnostic markers such as an elevated AG or measured serum methanol concentration, and the patient had no visual sequelae, despite being later confirmed to have had a critical level of exposure.

In our case, serum methanol levels were not measurable at the time of presentation. However, the patient’s urine methanol concentration was 1370 mg/L. Based on the urine-to-serum ratio (0.816) reported by Hovda et al. [4], this corresponds to an estimated serum methanol concentration of approximately 1680 mg/L - clearly within the fatal range. Despite this, the patient exhibited a normal AG and no visual symptoms.

In addition, the patient presented with marked respiratory acidosis (pH 7.187, pCO_2_ 71.1 mmHg), which was likely related to central hypoventilation caused by flunitrazepam overdose. The absence of metabolic acidosis despite a toxic methanol exposure may be explained by several factors. First, the patient presented relatively early, before sufficient accumulation of formic acid. Second, the ingested solution contained approximately 5% ethanol in addition to methanol, which may have competitively inhibited alcohol dehydrogenase and delayed the production of toxic metabolites. Finally, prompt gastric evacuation may have reduced the amount of methanol and flunitrazepam absorbed, as suggested by the presence of blue, granular gastric contents in the aspirate. This striking discrepancy emphasizes the limitations of relying solely on conventional diagnostic markers. It underscores the importance of tailoring therapy not only to pathophysiology but also to available resources and clinical urgency.

This case illustrates that early clinical judgment and decisive intervention may be critical in preventing deterioration, even in the absence of typical diagnostic markers such as an elevated AG or measurable serum methanol levels. Our decision to initiate CRRT and ethanol therapy was based solely on clinical suspicion, supported by circumstantial evidence and early non-specific symptoms.

The choice of ethanol rather than fomepizole was driven by local unavailability of fomepizole at the time, while ethanol could be administered immediately at the bedside. Although ethanol carries risks of hypoglycemia and central nervous system depression, its benefit in preventing formate formation outweighed these concerns under close ICU monitoring. Likewise, while intermittent hemodialysis (IHD) is generally the modality of choice for methanol elimination [10], CRRT was selected because it was more readily accessible in our ICU and could be initiated without delay. The risk of slower clearance with CRRT was balanced against the advantage of timely initiation, and prior cohort studies have shown comparable outcomes between CRRT and IHD when adjusted for severity [11]. This risk-benefit assessment underscores the importance of tailoring therapy to available resources and clinical urgency. The favorable outcome, without any visual or neurologic sequelae, demonstrates the critical importance of timely empirical treatment in suspected methanol poisoning, especially in settings where diagnostic resources are limited [3].

This report has several limitations. The serum methanol level was not measured, and the diagnosis and treatment were based solely on clinical suspicion, without the aid of quantitative markers at the time of presentation. Although a high urinary methanol concentration was later confirmed, such surrogate data may be affected by the timing of ingestion, renal clearance, and individual metabolic variability [6].

In addition, although IHD is generally considered the standard of care for extracorporeal methanol elimination [10], CRRT was used in this case because it was immediately available in the ICU. This pragmatic choice allowed the timely initiation of therapy, though its clearance rate is slower than IHD. To improve decision-making in resource-limited or urgent settings, future research should focus on developing practical algorithms that integrate clinical features such as unexplained metabolic acidosis, high AG/OG, or surrogate markers like urinary methanol, while taking into account the local availability of treatment modalities.

## Conclusions

This case demonstrates that timely empirical treatment based on clinical suspicion can be life-saving in methanol poisoning, even in the absence of typical diagnostic markers or access to standard antidotal therapy. Our experience underscores the value of early clinical decision-making and shows that appropriate intervention is achievable even in resource-limited settings. In this instance, treatment was initiated despite the absence of objective confirmatory evidence, relying solely on the patient’s account and the potential lethality if her statement was correct. This approach highlights that, in suspected toxic alcohol ingestions, the risk of delayed intervention may outweigh the risk of overtreatment. We hope this case encourages clinicians to trust their clinical judgment and act decisively when managing suspected toxic alcohol ingestions, especially in settings where confirmatory tools may be unavailable. This structured reasoning process may also serve as a reference for clinicians confronted with similar diagnostic uncertainty.
